# Upregulated IL-21 and IL-21 receptor expression is involved in experimental autoimmune uveitis (EAU)

**Published:** 2009-12-31

**Authors:** Lan Liu, Yongfeng Xu, Jianyong Wang, Huiyan Li

**Affiliations:** 1 Department of Ophthalmology, First Affiliated Hospital, College of Medicine, Zhejiang University, Hangzhou, P.R. China; 2 Department of Neurology, Second Affiliated Hospital, School of Medicine, Zhejiang University, Hangzhou, P.R. China

## Abstract

**Purpose:**

Interleukin (IL)-21 has recently been shown to play a vital role in the development of many autoimmune diseases. Our study is designed to investigate the alteration and possible function of IL-21 in the development of an experimental autoimmune uveitis (EAU) model.

**Methods:**

EAU was induced in B10.RIII mice by subcutaneous injection of interphotoreceptor retinoid-binding protein (IRBP) 161–180 emulsified with complete Freund’s adjuvant (CFA) and evaluated by clinical and histopathologic observation. *IL-21* and *IL-21R* mRNA expressions in cells of draining lymph node (DLN) and spleen in EAU and control mice were determined by reverse transcription-PCR. The frequencies of interleukin-21 receptor positive cells were also examined using flow cytometry. IL-17 levels in the supernatant of the cell culture upon IL-21 stimulation were assayed by enzyme-linked immunosorbent assay.

**Results:**

Results showed that EAU was successfully induced by IRBP161–180. Expression of *IL-21* mRNA was significantly increased in cells of DLN and spleen in EAU compared with recovery phase mice and normal controls. *IL-21R* was also found upregulated in DLN and spleen cells of EAU mice by reverse transcription-PCR and flow cytometry. Cells in EAU cultured with IL-21 combined with transforming growth factor-β induced increased production of IL-17.

**Conclusion:**

The findings revealed that increased *IL-21* and *IL-21R* expression may be involved in the development of EAU, possibly by promoting IL-17 secretion.

## Introduction

Human uveitis is a group of ocular inflammatory disorders that affects the uvea, retina, vessels, and vitreous. Uveitis and its associated complications cause the progressive destruction of the eye and eventually result in blindness. Experimental autoimmune uveitis (EAU) is a well-established animal model for the human disease [1]. T helper (Th)1 cell-mediated response was considered in the early years, to play a critical role in the pathogenesis of uveitis [2]. However, recent studies have demonstrated that interferon (IFN)-γ is not the only key cytokine responsible. Interleukin (IL)-12 has protective effects in the development of diseases in the EAU model [3,4], while IL-17-producing CD4^+^ T cells (Th17 cells, distinct from classic Th1 and Th2 cells) exerts more pronounced functions in autoimmune diseases [5-7]. Upregulation of the IL-17–IL-23 pathway has also been reported to play an important role in the pathogenesis of human autoimmune diseases, but its precise mechanisms in autoimmune uveitis remain unclear.

IL-21 is a novel pro-inflammatory cytokine that was recently found to contribute to immune responses in the Th17 pathways. It was first identified in 2000 as a new member of the type I four-α-helical-bundle cytokine family; its receptor shares the common γ chain and signals via the Janus kinase (JAK)/ Signal Transducers and Activators of Transcription (STAT) pathway [8,9]. In addition, IL-21 has been shown to be mainly produced by activated CD4^+^ T cells, but its receptor has been detected on several different cell types. Researchers have found that IL-21 has broad effects on both innate and adaptive immunity; it induces the proliferation and differentiation of T and B lymphoid cells and increases the activity of CD8^+^ T and natural killer cells [10,11]. Recently IL-21 has been characterized as being closely related to Th17 cells; it promotes the differentiation of Th17 cells and augments IL-17 production in vitro [12,13]. The pro-inflammatory functions of IL-21 are also associated with many autoimmune disease models, including experimental autoimmune encephalomayelitis, adjuvant-induced arthritis and experimental colitis [12,14,15]. However, whether IL-21 is involved in the development of uveitis, a typical autoimmune disease, and whether it can regulate IL-17 production in EAU, are still unknown.

This study was, therefore, designed to investigate the alteration and possible function of IL-21 in the development of the EAU model. Our results showed that the expression of IL-21 was markedly increased in both splenocytes and draining lymph node (DLN) cells of EAU mice compared to that in the normal controls and the recovery phase. The expression of the IL-21 receptor (IL-21R) was also found significantly elevated in EAU mice in both mRNA and protein levels. Recombinant IL-21 could promote the increase in IL-17 production by splenocytes and DLN cells of EAU mice. in vitro These data suggest that upregulated IL-21 production may play an active role in the development of inflammation in EAU mice.

## Methods

### Mice

B10.RIII mice were purchased from the Jackson Laboratory (Bar Harbor, ME). Mice were housed in a specific pathogen-free facility at the animal center. Animal experiments were performed at the age of 6–8 weeks according to the ARVO statement for the Use of Animals in Ophthalmic and Vision Research. All animal procedures were approved by the institution’s animal care board.

### Experimental autoimmune uveitis induction and scoring

Human interphotoreceptor retinoid-binding protein (IRBP) peptide 161–180 (SGIPYIISYLHPGNTILHVD) was synthesized by the Shanghai Shenggong company (Shanghai, China). Mice were immunized with a subcutaneous injection of human IRBP peptide 161–180 (50 μg/mouse), emulsified (1:1, vol/vol) with complete Freund’s adjuvant (CFA; Sigma-Aldrich, St. Louis, MO). Pertussis toxin (Sigma-Aldrich), 1 μg in 0.1 ml, was concurrently injected intraperitoneally. Mice were examined by slit lamp and direct funduscopy for clinical signs. For histopathologic evaluation, eyes were enuculated, fixed in 10% phosphate-buffered formaldehyde, dehydrated, and embedded in methacrylate. Then 5-µm sections were cut through the pupillary–optic nerve plane and stained with hematoxylin and eosin (H&E; Guangzhou, China). Disease scores were evaluated on the extent of inflammation and tissue damage by histopathology, as described [16]. Briefly, retinal perivascular infiltration and monocytic infiltration in the vitreous were scored as 1; granuloma formation, the presence of occluded retinal vasculitis, photoreceptor folds, serous detachment and loss of photoreceptor were scored as 2; formation of Dalen-Fuchs nodules, and development of subretinal neovascularization were scored as 3 and 4, according to the number and the size of the lesions. Mice (Week 5-6 or more after immunization) showed no inflammation by clinic and histopathological observation were considered as mice of recovery phase. Mice that were immunized with only CFA were used as controls.

### Cell isolation and culture

Lymphocytes were harvested from the spleen and DLN, using Ficoll-Hypaque density gradient centrifugation. Cells were resuspended at a density of 2×10^6^ cells/ml in RPMI  1640 medium (Gibco, Grand Island, NY) containing L-glutamine (2 mM), penicillin/streptomycin (100 U/ml), and 10% fetal calf serum. For the cytokine assay cells were cultured with or without recombinant mouse interleukin-21 (rmIL-21; 100 ng/ml) and recombinant transforming growth factor-β (rTGF-β; 5 ng/ml; both from R&D Systems, Minneapolis, MN) for 72 h at 37 °C, 100% humidity, and 5% CO2.

### Reverse transcription polymerase chain reaction

Total RNA was extracted from the splenocytes and DLN cells, using the RNAprep Cell kit (Tiangen Biotech, Beijing, China), according to the manufacturer’s instructions. Briefly, pellet of 5×10^6^ cells was mixed with 350 μl lysate, transferred into a filtration column, centrifuged 12,000 rpm for 30 s. Then the pellet was washed with 70% ethanol, deproteinization water in an absorption column. The absorption column was resuspended with 50 μl RNAase-free water and centrifuged 12,000 rpm for 2 min. Total RNA was collected in a RNAase-free tube. Reverse transcription of RNA into cDNA was performed using the Superscript III Reverse Transcriptase system (Invitrogen). The specific primers used for *β-actin*, *IL-21*, and *IL-21R* used in this study were: β-actin sense: 5′-GTC CCT CAC CCT CCC AAA AG-3′, antisense: 5′-GCT GCC TCA ACA CCT CAA CCC-3′. IL-21 sense: 5′-TCA TCA TTG ACC TCG TGG CCC-3′, antisense: 5′-ATC GTA CTT CTC CAC TTG CAA TCC C-3′, IL-21R sense: 5′-CTC CCC CCT TGA ACG TGA CT-3′, antisense: 5′-TTG CCC CTC AGC ACG TAG TT-3′. The mRNA expression levels of each sample were normalized to the levels of *β-actin*. A total amount of 5 μl PCR product of each sample was electrophoresed in the 2% agarose gels. The density of bands was quantified by densitometry and analyzed with Genetools software (Synoptics Ltd, Cambridge, UK).

### Flow cytometry

The isolated cells were stained with anti-CD3, anti-CD4, anti-CD8, and anti-IL-21R antibodies (eBioscience, SanDiego, CA) for 0.5 h for the analysis of surface markers. Cells were then washed three times and suspended in PBS. fluorescence-activated cell sorting (FACS) analysis was performed using FACS Calibur and CellQuest software (BD Biosciences, Shanghai, China ).

### Cytokines in enzyme-linked immunosorbent assay

Supernatants were collected from cell cultures with different stimulation, as mentioned above. IL-17 was measured using the mouse IL-17 DuoSet enzyme-linked immunosorbent assay (ELISA) development kit (R&D Systems), with a detection limit of 5.6 pg/ml.

### Statistics

The data were analyzed using SPSS 10.0 (SPSS, Inc. Chicago, IL). The independent Student *t*-test was used to assess differences between groups. Results were expressed as mean±standard deviation, and p<0.05 was considered significant.

## Results

### Induction of the experimental autoimmune uveitis model

**Figure 1:**
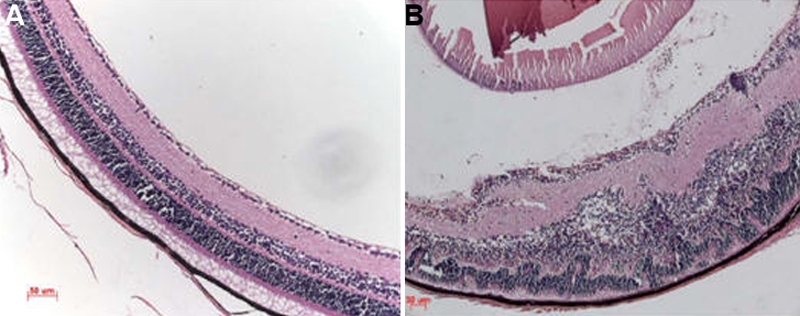
Histopathologic features of the eyes of experimental autoimmune uveitis (EAU) mice. Eyes were enucleated from EAU and complete Freund’s adjuvant (CFA) control mice on day 14 after immunization. Sections (5 µm) were cut through the pupillary–optic nerve plane and stained with hematoxylin and eosin. **A**: Eye of CFA controls. The retina layers are well ordered and preserved. **B**: Eye of EAU mice. The normal structure of the retina is disorganized. Inflammatory cells infiltrate the ciliary body, choroid, vitreous and retina. Vasculitis, granuloma formation, and retinal folding are visible, and the photoreceptor layer is damaged. The scale bar=50 µm.

The EAU model was induced by immunization of IRBP peptide 161–180 and evaluated by clinical observation and H&E staining of the eye section. Flare and cells in the anterior chamber, and vasculitis and choroiretinal lesions in the fundus were observed in the EAU mice. Histopathologic examination showed inflammatory cell infiltration of the ciliary body, choroid, vitreous and retina, vasculitis, granuloma formation, retinal folding, detachment, and photoreceptor damage (Figure 1B). There were no inflammatory signs observed by clinical and histopathologic evaluation of mice during the recovery phase (5–6 weeks after immunization). Control mice (immunized with CFA only) showed no inflammation clinically or histopathologically (Figure 1A).

### *Interleukin-21* expression is increased in splenocytes and draining lymph node cells of experimental autoimmune uveitis mice

**Figure 2:**
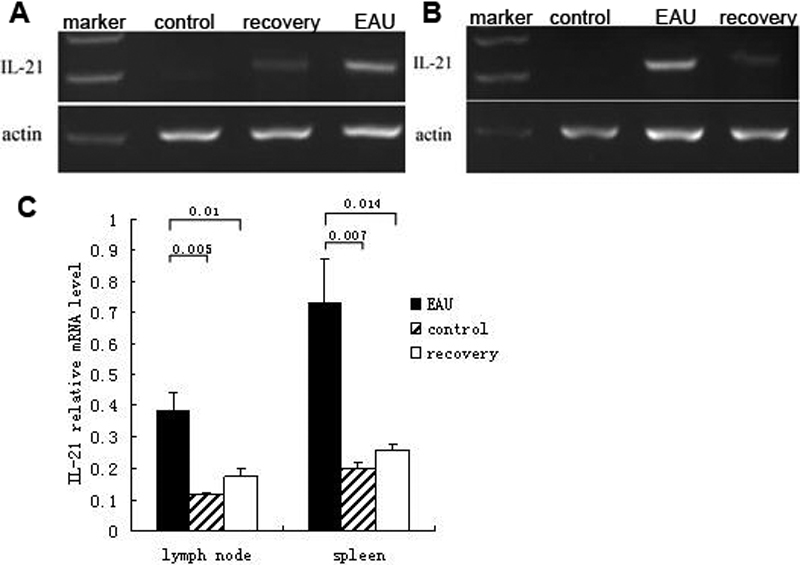
Reverse transcription-PCR analysis of interleukin-21 in the cells of spleen and draining lymph node in experimental autoimmune uveitis, recovery phase and control mice. mRNA of IL-21 in splenocytes and draining lymph node (DLN) cells of EAU, recovery phase mice, and control mice were extracted and measured through RT–PCR. **A**: *IL-21* mRNA of DLN cells in EAU, recovery phase mice, and control mice. **B**: *IL-21* mRNA of spleen cells in EAU, recovery phase mice, and control mice. **C**: The relative *IL-21* mRNA levels were determined as the ratio of the *IL-21* PCR product over the *β-actin* product. Data are presented as mean±standard deviation; n=6 per group.

*IL-21* mRNAs in splenocytes and DLN cells of EAU mice, recovery phase mice, and control mice were extracted and measured using reverse transcriptase (RT)-PCR. *IL-21* levels were presented by the intensity ratio of the *IL-21* mRNA band divided by *β-actin* mRNA band. Results showed that mRNA expressions of *IL-21* were significantly increased in DLN cells of EAU mice (n=6) compared with recovery phase mice (n=6, p=0.01) and normal controls (n=6, p=0.005; Figure 2A,C). *IL-21* levels in the splenocytes of EAU mice (n=6) were also markedly higher than that of recovery phase mice (n=6, p=0.014) and normal controls (n=6, p=0.007; Figure 2B,C).

### Interleukin-21 receptor expression is elevated in splenocytes and draining lymph node cells of experimental autoimmune uveitis mice

**Figure 3:**
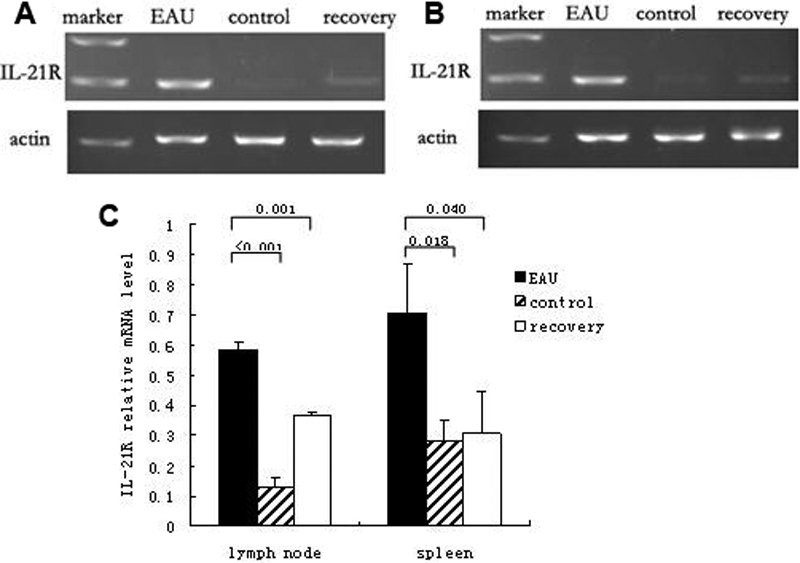
Reverse transcription-PCR analysis of interleukin-21 receptor in the cells of spleen and draining lymph node in experimental autoimmune uveitis, recovery phase and control mice. mRNA of *IL-21R* in splenocytes and draining lymph node (DLN) cells of EAU, recovery phase mice, and control mice were extracted and measured through RT–PCR. **A**: *IL-21R* mRNA of DLN cells in EAU, recovery phase mice, and control mice. **B**: *IL-21R* mRNA of spleen cells in EAU, recovery phase mice, and control mice. **C**: The relative *IL-21* mRNA levels were determined as the ratio of the *IL-21* PCR product over the *β-actin* product. Data are presented as mean±standard deviation; n=6 per group.

The expressions of IL-21R (*IL-21R*) in splenocytes and DLN cells were detected by RT-PCR. Significantly increased *IL-21R* expressions were found in DLN cells of EAU mice compared with recovery phase mice (p=0.001) and normal controls (p<0.001; Figure 3A,C). The results also showed that *IL-21* expressions were markedly elevated in the splenocytes of EAU mice (p=0.04 compared to recovery mice [p=0.018] and normal controls; Figure 3B,C).

**Figure 4:**
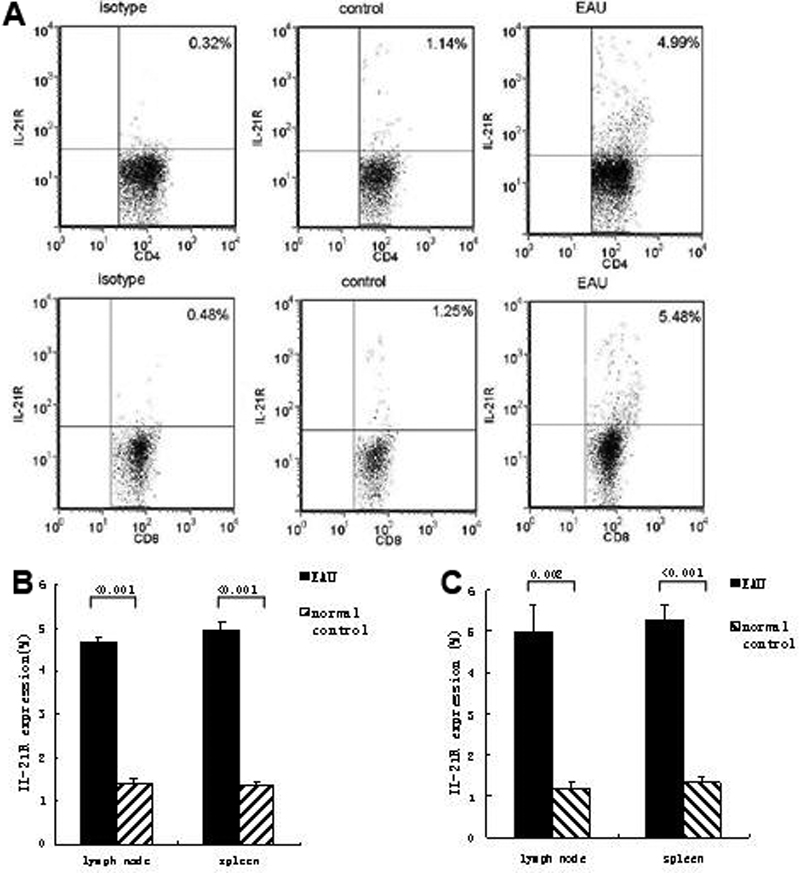
The percentage of interleukin-21 receptor positive cells from draining lymph node of experimental autoimmune uveitis and control mice. The isolated DLN cells were stained with anti-CD4, anti-CD8, and anti-IL-21R Ab for 0.5 h for the analysis of surface markers. **A**: Dot plots of IL-21R^+^ cells in CD4^+^ and CD8^+^ cells of EAU and control mice. Data shown are representative of six independent experiments. **B**: Percentages of IL-21R^+^ cells in CD4^+^ T cells of EAU and control mice. **C**: Percentages of IL-21R^+^ cells in CD8^+^ T cells. Data are presented as mean±standard deviation; n=6 per group.

We further investigated the expressions of IL-21R at the single cell level using the method of flow cytometry. As showed in Figure 4, IL-21R expressions were significantly increased in both CD4^+^ and CD8^+^ T cells in DLN of EAU mice compared with that of normal controls (p<0.001 in CD4^+^T cells; p=0.002 in CD8^+^T cells). A similar result was observed in the splenocytes. IL-21R expressions in the splenocytes were prominently higher in EAU mice than in control mice (p<0.001 in CD4^+^T cells; p<0.001 in CD8^+^T cells).

### Recombinant interleukin-21 combined with transforming growth factor-β increases interleukin-17 secretion in experimental autoimmune uveitis

**Figure 5:**
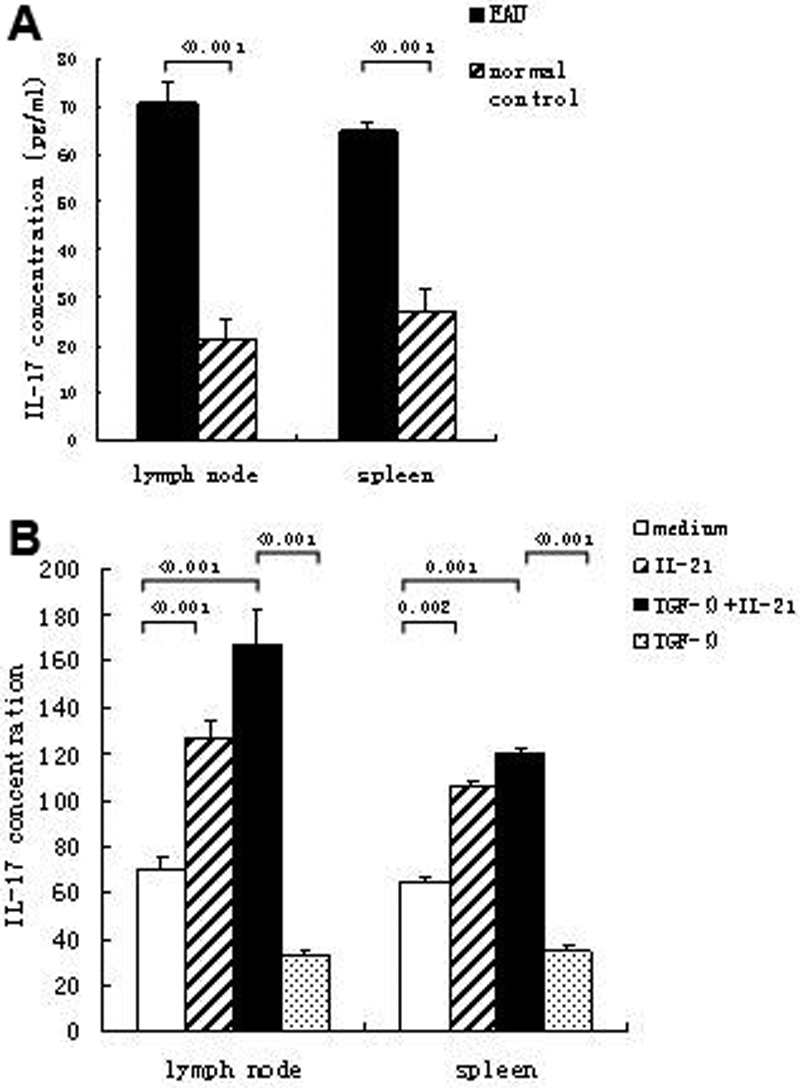
Interleukin-17 production in the culture supernatant of experimental autoimmune uveitis and control mice. Cells from DLN and spleen were cultured with or without stimulation. The concentration of IL-17 in the supernatant was detected by ELISA. **A**: IL-17 production by DLN and spleen cells in EAU and control mice without stimulation. **B**: IL-17 production by DLN and spleen cells after IL-21 combined with or without TGF-β stimulation. Data are presented as mean±standard deviation; n=6 per group.

The previous results suggested that IL-21 might be involved in the development of the immune reaction in EAU. Therefore, we further investigated whether IL-21 could influence the production of IL-17, a vital inflammatory cytokine in the pathogenesis of uveitis. Cells were cultured with a stimulation of recombinant IL-21 combined with transforming growth factor (TGF)-β. Production of IL-17 in the supernatant was detected by ELISA. A markedly higher concentration of IL-17 in the supernatant of the cell culture was found in EAU mice compared to normal controls without any stimulation (p<0.001 in both DLN and spleen; Figure 5A). Stimulation of recombinant IL-21 significantly upregulated IL-17 secretion in DLN cells of EAU mice (p<0.001; Figure 5B). IL-17 production was also significantly increased in the supernatant of the DLN cell cultures stimulated with IL-21 combined with TGF-β compared to only TGF-β stimulation (p<0.001; Figure 5B). Similar to DLN cells, the concentration of IL-17 in the cell culturesof splenocytes was significantly elevated after stimulation of IL-21 (p=0.002) or combined with TGF-β (p<0.001).

## Discussion

Uveitis is considered a typical T-cell mediated, organ-specific, autoimmune disease, but its etiology and pathology are still elusive [17]. In this study, we explored the involvement of IL-21 in the pathogenesis of EAU, given that IL-21 acts as an essential cytokine in immune responses and plays important roles in many autoimmune diseases [18]. Our results showed that the expression of IL-21 and IL-21R were significantly higher in both splenocytes and DLN cells of EAU mice compared with those in recovery phase mice and controls. In vitro recombinant mouse IL-21 combined with or without TGF-β could induce markedly increased IL-17 secretion in cell cultures of splenocytes and DLN cells of EAU mice. These results suggest that IL-21 may be actively involved in promoting the development of EAU.

It has been demonstrated recently that IL-21 plays a pivotal role in either initial or acquired immunity and is closely associated with several autoimmune diseases, such as multiple sclerosis and inflammatory bowl disease [12,14]. Administration of IL-21 promoted the inflammatory influx and increased the severity of disease, and IL-21-deficient mice showed markedly reduced disease score [13]. To further investigate its function, we analyzed the role of IL-21 in EAU, a typical T-cell-mediated autoimmune disease. First we examined the expression of IL-21 in EAU mice and controls. *IL-21* mRNA levels in the DLN cells of EAU mice were upregulated compared with recovery phase mice and controls. We found a similar augmentation in the splenocytes of EAU. These results indicate that the increased expression of *IL-21* is associated with the development of EAU.

IL-21 executes its function through its receptor, which is expressed by several types of immune cells. A higher expression of IL-21R was also found in several autoimmune disorders, and blockade of the IL-21R pathway resulted in amelioration of the disease [19,20]. Therefore, we examined whether IL-21R expressions in DLN and spleen cells of EAU mice are also elevated. Results showed that IL-21R expressions are significantly increased in both DLN and spleen cells of EAU mice at the mRNA level compared to recovery phase mice and controls. Elevated IL-21R expression was also detected in EAU at the protein level by flow cytometry analysis. There was a markedly higher IL-21R expression in either CD4^+^ or CD8^+^ cells of EAU mice compared with controls, and we obtained similar results for both DLN and spleen cells. Other studies have shown that IL-21R is upregulated on T cells upon activation [9]. Thus, the results that CD4^+^ and CD8^+^ T cells express high levels of IL-21R suggest that these cells may be the potential target cells of IL-21 in EAU mice.

The mechanism of IL-21 function and its specific role in Th1/Th2/Th17 cell differentiation and related response remains to be clarified. Recent studies have shown that IL-21 is highly expressed by Th17 cells and could promote the differentiation of Th17 cells in vitro and in vivo [21-23]. In view of the crucial effects of IL-17 in the pathogenesis of autoimmune diseases, including uveitis [24], we investigated the influence of IL-21 on IL-17 production in the EAU model. IL-17 was first found significantly increased in the DLN and spleen cells of EAU mice compared to controls without stimulation. This suggests that IL-17 plays an important role in the development of autoimmune uveitis. The effects of IL-21 on IL-17 production were observed using methods similar to those used by other authors [12,13]. Our results showed that IL-21 would induce IL-17 secretion in the DLN and spleen cells of EAU mice. IL-21 combined with TGF-β stimulation promoted a significantly increased IL-21 production in the EAU cells.

The theories concerning the role of IL-21 in Th1, Th2, and Th17 responses are still controversial. Studies have demonstrated that IL-21 is essential for the differentiation of Th17 cells and plays a crucial role in the pathogenesis of several autoimmune diseases [12,13,21]. There is also a report that IL-21 is not essential for Th17 expansion in EAE models [25]. This discrepancy might be because of the different model and genetic backgrounds of the mouse lines used for the two studies. A recent study has also shown that IL-21 production is not restricted to CD4^+^ T cells; natural killer T cells are also potent producers of IL-21 [26]. Studies of the detailed mechanisms of IL-21 function in EAU, including the cellular source of IL-21, will be performed in the near future. Inhibition experiments using anti-IL-21 antibody or IL-21 knockout mice should also be done to further identify the effects of IL-21 in EAU.

In summary, our study showed that increased IL-21 production and elevated IL-21R expression may be associated with the development of autoimmune uveitis in mice. Our results showed that IL-21 or combined with TGF-β would induce increased IL-17 production. With the suggestion that the IL-21–IL-21R pathway may be involved in the pathogenesis of autoimmune uveitis, we propose a possible therapeutic approach for autoimmune uveitis through the manipulation of IL-21 secretion.
